# An application of Harrison's system theory model to spark a rapid telehealth expansion in the time of COVID‐19

**DOI:** 10.1002/lrh2.10239

**Published:** 2020-07-26

**Authors:** Jonathan C. Touson, Namita Azad, Corinne Depue, Timothy Crimmins, Rosalie Long

**Affiliations:** ^1^ Columbia University Irving Medical Center New York New York USA

**Keywords:** COVID‐19, Harrison, system theory, telehealth

## Abstract

**Introduction:**

In response to the COVID‐19 pandemic, health systems had to quickly adopt a process for enabling targeted and patient‐centered care delivery. This case study describes the utilization of Harrison's open‐systems model to create an approach for rapid adoption of existing telehealth technologies in a large scale academic medical center.

**Methods:**

An internal group of organizational developers, was enlisted to enable this effort. Local networks were employed and organized into focus groups to rapidly assess and address barriers to adoption and informal interviews with executive leadership were conducted to align organizational goals. Interventions include rapid deployment of focused and data driven provider, staff and patient support bolstered by effective communication and resource management.

**Results:**

There was an increase in the number of patient portal activation codes by 75% during the month of March. The number of activation codes generated expectedly decreased in April as many patients now had activated patient portals. The video visit volume as a result of provider self‐scheduling increased went from a baseline of 0 to over 600 clinical visits.

**Discussion:**

Experienced organizational development programs can facilitate adoption of change. The faculty practice of CUIMC has years of experience with supporting wide scale operational change centered on technology. In this case, providing engaged networks with tailored content that is focused on the process and available technology promoted rapid adoption and optimization.

**Conclusion:**

In the setting of profound external pressure, experience with the ability to focus on tailoring training and support to the culture of the organization helped to rapidly increase the availability and success of telehealth visits for a large scale academic medical center.

## 1 INTRODUCTION

The open‐systems approach was first applied to organizations by Katz and Kahn,^1^ who adapted the General Systems Theory to organizational behavior and how organizations react, adjust, and re‐equilibrate to changing conditions from internal or external sources. This approach identifies organizational behavior by mapping the repeated cycles of input, throughput, output, and feedback between an organization and its external environment. Systems receive input from the environment either as information or in the form of resources. The systems then process the input internally, by applying transformational capabilities to absorb the new input, and release outputs into the environment, in order to restore equilibrium or improvements to the environment. The system then seeks feedback from the environment to determine whether the output was effective in restoring equilibrium, improving performance, or further adjustments are needed.

**FIGURE 1:**
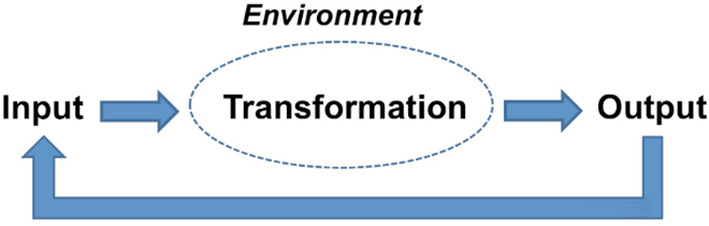
Open‐systems model

Harrison^2^ proposes that open systems models are organic in nature, as they involve interactions between internal elements and the external environment. Historically, other organizational theories have overlooked environmental influences on the organization, which they consider a closed system. Open systems models, in Harrison's view, involve the cyclical elements of input, transformation, and output within organizations. The output from one cycle becomes the input for the next cycle, and the entire process takes place in the context of a particular environment (Figure 1).

**FIGURE 2:**
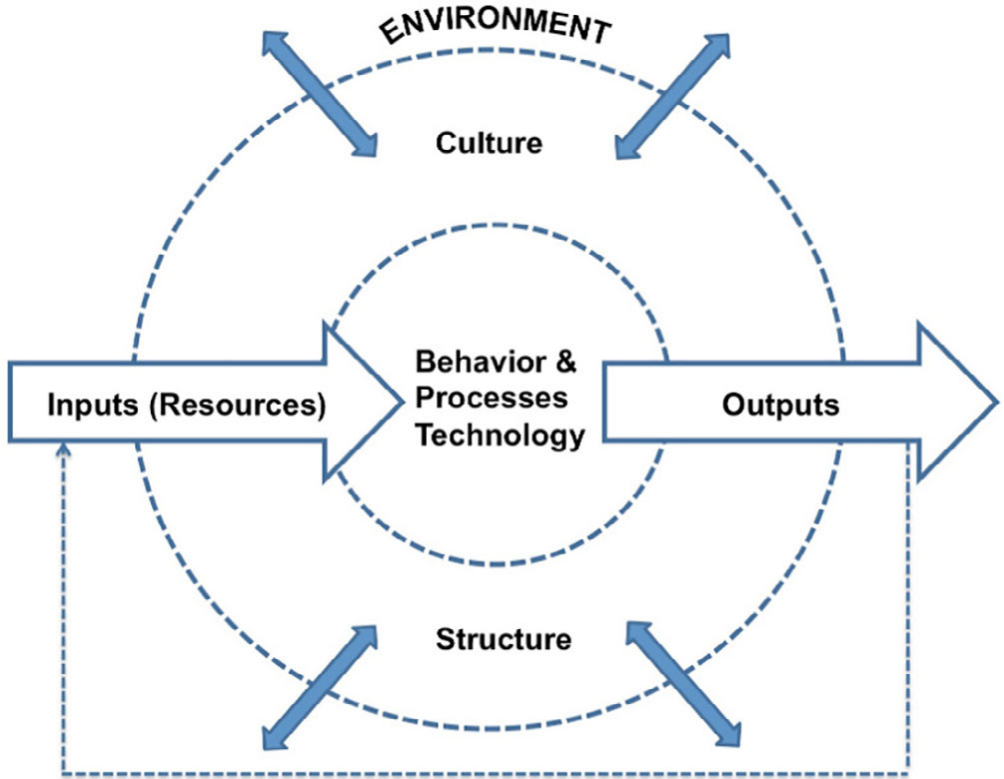
Harrison model

In a more detailed representation of this model Harrison^2^ shows how the dynamic relationship between the system's components and its layers are critical to be diagnosed in order to understand how the input, transformation mechanism, and output, as well as their relationships with the environment interact with each other and how modifications in one of those could potentially redefine the others (Figure 2).

Like other healthcare systems around the world, the largest environmental factor that the Columbia University Irving Medical Center (CUIMC) is facing is the global pandemic^3^ of the novel coronavirus, COVID‐19. The emergence and rapid spread of this virus has placed unprecedented amounts of pressure on CUIMC's workforces and resources. The impact of this environmental input was studied through Harrison's^2^ model to design and deploy an approach for a rapid telemedicine expansion (available technology) to redefine, improve, and adjust CUIMC's output: Patient care.

## 2 QUESTIONS OF INTEREST

The questions of interest discussed in this paper are:

1. Can the utilization of telehealth technologies in an organization (behavior), be rapidly evolved and expanded by incorporating change management methodologies? This required a renewed implementation effort (process) to adapt to a new environmental need (COVID‐19 pandemic).
2. Does engagement and adoption of new workflows increase when layers and components of the system (structure) are activated using culturally aligned initiatives? This included identifying the substructures of the organization and focusing on targeting engagement from their leaders to spark adoption. This approach needed to support the culture of a large academic medical center to more effectively facilitate adoption of the new processes.

## 3 MATERIALS AND METHODS

CUIMC has an established Organization Effectiveness and Optimization team whose primary objective is to enable the organization to proactively meet strategic goals by designing targeted, data‐driven, and cost‐effective interventions at the people, process and technology levels. As internal Organization Development practitioners, the group leveraged the existing layers of the organization and performed an assessment of the current state with frontline staff. Departments were prioritized based on patient volume and revenue impact. The departments were asked to participate in a set of virtual focus groups^4^ where audiences were individual department administrators and practice managers. These sessions were facilitated with a structured approach using a series of pre‐determined questions to assess the respective department's level of adoption, barriers and success cases of video visits. The responses indicated gaps in understanding specifically the patient based workflow, scheduling new and converting existing in person visits to video visits and the provider workflow. In collaboration with the internal training team, a set of education material, targeted towards providers and schedulers, was created and collated to close knowledge gaps that were assessed.

In addition, informal and unstructured interviews with the Chief Medical Information Officer and the Associate Chief Medical Information Officer were conducted for input on gaps in adoption across the organization, the inner environment. With guidance from the top‐level management and information gathered from the front‐line focus groups, another gap was discovered; the ability to adopt the workflow for different staffing models. With a deep understanding of the culture of the organization, a need was identified to ensure all content created was easily consumable. Two education topics were targeted, and the format of eLearning modules was used to present them:

1. Provider self‐scheduling workflow.
2. End‐to‐end video telehealth visit workflow inclusive of scheduler, provider and patient sub‐processes.

Both workflows support a variety of staffing models and allow transparency of the entire workflow so clinical practices can build redundancies. Flexible staffing models were key in our rapid workflow adoption. Each specialty, department and practice is unique. Targeted education materials and varied communication channels removed barriers that could impact the success of such a rapid transformation. This content was pushed out to the respective audiences using the existing learning management system, making it quickly accessible, and was also posted on an internal website. To further enhance adoption of these workflows, one‐on‐one training sessions were made available to care teams. These sessions reviewed the end‐to‐end workflow, common billing and technical concerns, and recommendations on distribution of resources to ensure the patient experience was maintained. Monitoring the volume of video visits conducted in the coming weeks would measure the success of these initiatives.

Patients are a critical piece of a healthcare organization's system and patient utilization of the telehealth technology needed to be optimized. Other groups within CUIMC had already made available numerous instructional materials and opened a telephone hotline to support patient questions. This effort was enhanced by including those materials and information in the end‐to‐end workflow video tutorial, focusing on how each staff member could help patients to activate their portal accounts. Practice staff were also provided recommendations on how to distribute resources to ensure there was a patient portal champion at each location who is a workflow expert to help patients troubleshoot issues or concerns. We would measure the success of these efforts by tracking the percentage of patients with a portal account activated.

The final step was the utilization of data to help foster a proactive culture. Patient lists that included data on a department's telehealth visit uptake—and where patients may have been lost to care—were created. Care teams utilize these lists to proactively reach out to this patient cohort to ensure they are engaged and help provide them with support.

**FIGURE 3:**
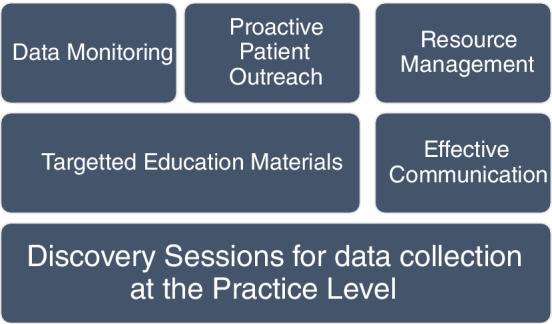
Multi‐layered methodology

This multilayered methodology is summarized in the diagram below (Figure 3). Each layer considers the technological, people and process inputs.

## 4 RESULTS

The expansion of the CUIMC telehealth strategy began on March 13th with deployment of the described methodology for gathering inputs via virtual focus groups, provider support sessions and communication channels. Throughout the next 2 weeks, a steady increase in volume of visits was registered which was a measure of success for the initiative (Figure 2). A key factor in achieving an increase in volume of video visits was implementing provider self‐scheduling, which enables providers to schedule patient visits independently. This was implemented on March 25th (via electronic learning module) and allowed practices to effectively conduct video visits with the minimum staff necessary (the scheduled visit provider).

To effectively remove barriers in telehealth adoption, our methodology focused on consistently gathering inputs to improve transformation support. We identified three key aspects of success in this transformation: staff training, patient readiness and effective communication. Our methodology for gathering inputs focused on implementing forums to identify barriers, then designing resolution plans and processes tailored to removing the specific barriers.

### 4.1 Virtual focus groups

From March 19‐March 27, over 20 virtual focus groups were conducted across specialties to share methodology and ascertain barriers to increasing outputs. Sessions were led by a focus group facilitator. Participants were asked a standard set of questions designed to assess the group's readiness and success in conducting video visits. Minutes were taken during the sessions and shared with participants, along with targeted training content. If issues/concerns could not be addressed in the meeting, the facilitator followed up with the participants outside of the session.

As highlighted in the methodology, ensuring patient readiness was critical. Activation of a patient portal account contributed to the success of a video visit. During virtual focus groups, change management methodologies were shared with the practices to reinforce the patient portal activation process and patient portal account set up. Aligning with previous trainings, in these sessions, offices were encouraged to prompt the patient to not only activate their account but to set up their application for the visit at multiple touchpoints prior to their visit. Throughout the month of March staff prioritized patient readiness, and the number of patient portal activation codes initiated by CUIMC staff jumped by almost 130 000 in the month of March (Figure 3). We consider this increase a successful outcome for our initiative as it measures the readiness of our patients for this transition. In April we saw an anticipated decline in generated activation codes as priorities shifted from readiness to execution of patient care.

### 4.2 Provider support sessions

The barriers and proposed methodology discussed in virtual focus groups fueled much of the content for the provider support sessions. These sessions were offered to each individual specialty for targeted support. The sessions were structured to present the resolution plans or processes designed as a result of the virtual focus groups but the sessions also allowed for flexibility in the event of the identification of new barriers during the sessions. Content discussed during the sessions varied from general training to billing and documentation support. These forums were vital in supporting our providers through this period of rapid change as they were the key agents of this transformation.

The virtual focus groups also identified a need for transparency among staff in the video visit workflow. The roles and responsibilities in conducting a successful in‐person visit were widely understood and there was a high level of transparency due to the sharing of physical space. To give all staff a holistic view of a successful telehealth visit, a complete end‐to‐end demonstration was developed and reviewed in the support sessions. This demonstration included all user roles involved in the execution of a video visit (ex. Scheduler, Provider and Patient).

### 4.3 Communication channels

**FIGURE 4:**
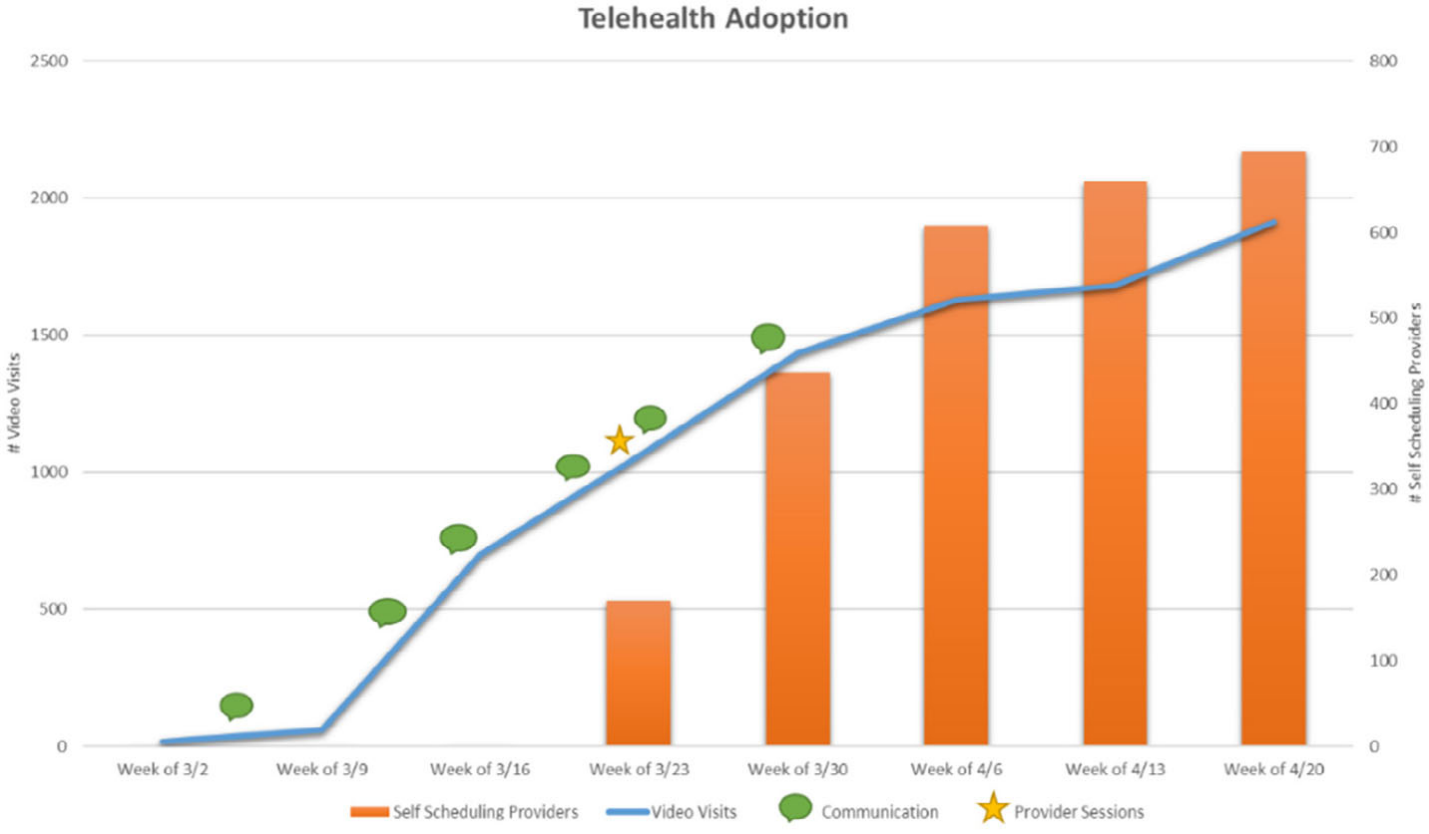
Results

During a time of reduced staffing and limited face‐to‐face communication, a need was identified for new and targeted communication channels. Communication during this time needed to be timely and consumable as regulatory changes were happening daily and training material on the new workflow was consistently being created or updated. Telehealth communication channels were implemented along with the overall strategy: daily huddle emails were distributed to operational leaders and email communications were sent directly to provider leaders in each specialty. The dates of the communication distributions are outlined on the Telehealth Visit Volume graph below (Figure 4). This graph displays the successful impact of the communication distributions on the increase of the volume of video visits.

## 5 DISCUSSION

The results of the implementation of the telehealth expansion strategy clearly indicates that when each system's sub‐structure receives content that is tailored to its role, focused on the process and available technology, they are quicker to adopt and optimize. The increase in telehealth visits once all the initiatives were rolled out proves that the Harrison model^2^ is effective for this type of environmental change. The data also supports that communication is more effective when delivered through the right targeted channel.

Throughout the Readiness and Provider Support sessions we identified several consistent barriers to the completion of a successful video visit that required the revision of material and reinforcement of some communication pieces. In some cases, targeted workarounds had to be put in place to facilitate the process and avoid delays on the implementation and adoption of the tool.

During the initial Epic go‐live for CUIMC in 2020, patient portal proxy activation workflows were reviewed in training and adopted across the organization. During this expansion of Telehealth workflows there was significant confusion about how proxy relationships impacted the Video Visit workflow for the patient.

Documentation and billing for video visits was identified as an area requiring additional support and communication. For example, few providers or administrators were aware that video visits should be documented and billed the same as an in‐office visit; the only variance is the inclusion of the required regulatory information for a telehealth visit in the progress note.

Due to the rapid change, there were technical and user issues that impacted the success of some video visits. This triggered the requirement for communication pertaining to workflows in failed video visit scenarios. This included guidance on billing in scenarios where the visit was disconnected prior to vs after fulfilling the E&M requirements. In addition, providers were offered guidance on billing for telephone visits in cases where patients did not have access to, or feel comfortable using, a smart device. While the patient portal video platform is the primary approach for telehealth visits, alternative video platforms and telephone calls can be used in select circumstances (ex. technical failure, patient unable to use portal). Communication and guidance was shard with providers, encouraging the transition to an alternative platform if the circumstances required. The issues outlined were consistently addressed using all the created channels.

## 6 LIMITATIONS

The Organization Development team rolled‐out these initiatives simultaneous with initiatives rolled‐out at the top management level. Due to limitations on data, direct and exact correlation between this initiative and the increase of telehealth visit volume cannot be determined. The assumption is that an unknown percentage of the increase and perhaps an improved rate of increase can be attributed to this initiative. Further, the Organization Development team did not have a predetermined process for engaging patients directly to assess their barriers and design interventions. We assume that by developing a direct patient engagement process, the team can be even more effective in supporting any required future and unknown adaptions to health care delivery.

## 7 CONCLUSION

COVID‐19 prompted many significant changes to the way healthcare is delivered worldwide. As other industries moved to virtual interactions, healthcare organizations were challenged with the obligation to continue to deliver patient care in a virtual capacity. With reliable technology, the measure of success throughout this change will depend on the readiness, training and support available for staff and patients. The strategy detailed in this case report outlines the methodology and model used to rapidly increase the availability and success of telehealth visits for a large scale academic medical center by focusing on tailoring training and support to the culture of the organization. Given that this strategy was supported by Harrison's open systems concepts of evaluating internal elements and accounting for external environment, it makes it easily replicable to other optimization and adoption initiatives.

## CONFLICT OF INTEREST

The authors affirm that they have no conflicts of interest.
